# Supplementary material for: Capsaicin camphor and caffeic acid reduce adipogenesis and promote lipolysis with TRPV1 involvement
Source: Sci Rep. 2025 Dec 2;15:43024. doi: 10.1038/s41598-025-31073-3 (PMC12675751; doi:10.1038/s41598-025-31073-3)
Supplement: Supplementary file 1 — Supplementary Material 1 [file 41598_2025_31073_MOESM1_ESM.docx]

**Supplementary Information**

**Capsaicin Camphor and Caffeic Acid Reduce Adipogenesis and Promote Lipolysis with TRPV1 Involvement**

**Bioactive compounds modulate lipid metabolism**

*Uzair Ali^1^, Franziska Wasner^1^, Jiajian Fan^1^, Martin Wabitsch^2^, Danijela Gregurec^1*^*

^1^Department of Chemistry and Pharmacy, University of Erlangen Nuremberg

^2^Department of Paediatrics and Adolescent Medicine University of Ulm

Correspondence: danijela.gregurec@fau.de


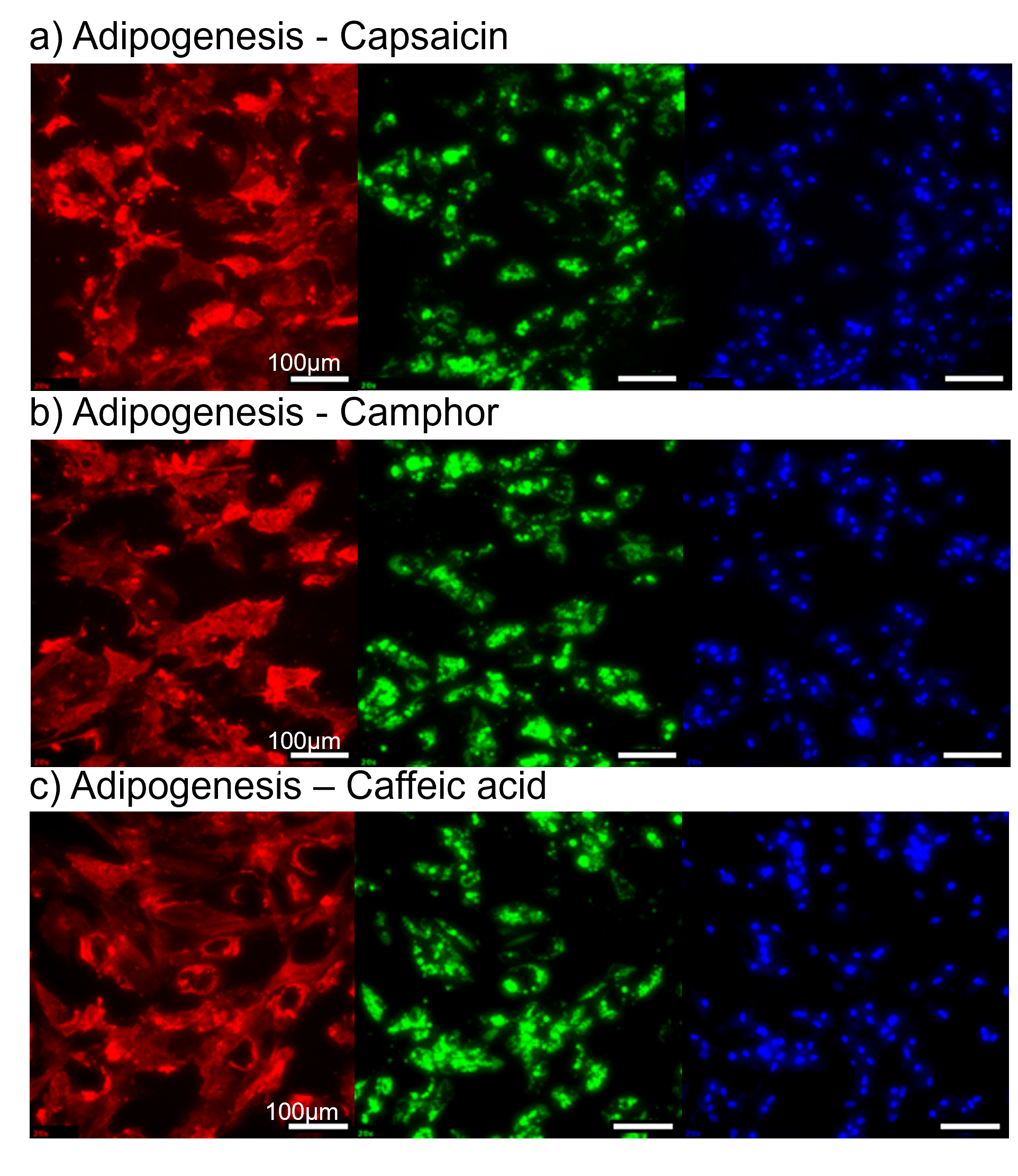


**Figure S1.** Immunofluorescence staining of SGBS adipocytes treated with capsaicin, camphor and caffeic acid and their combinations from day 6 to day 14 of differentiation (adipogenesis phase. Green, BODIPY 493,503 for neutral lipids. Red, TRPV1 immunofluorescence with Alexa Fluor 647 secondary. Blue, DAPI nuclei. Figure represents separated fluorescence channels that correspond to Figure 4 a in main manuscript.


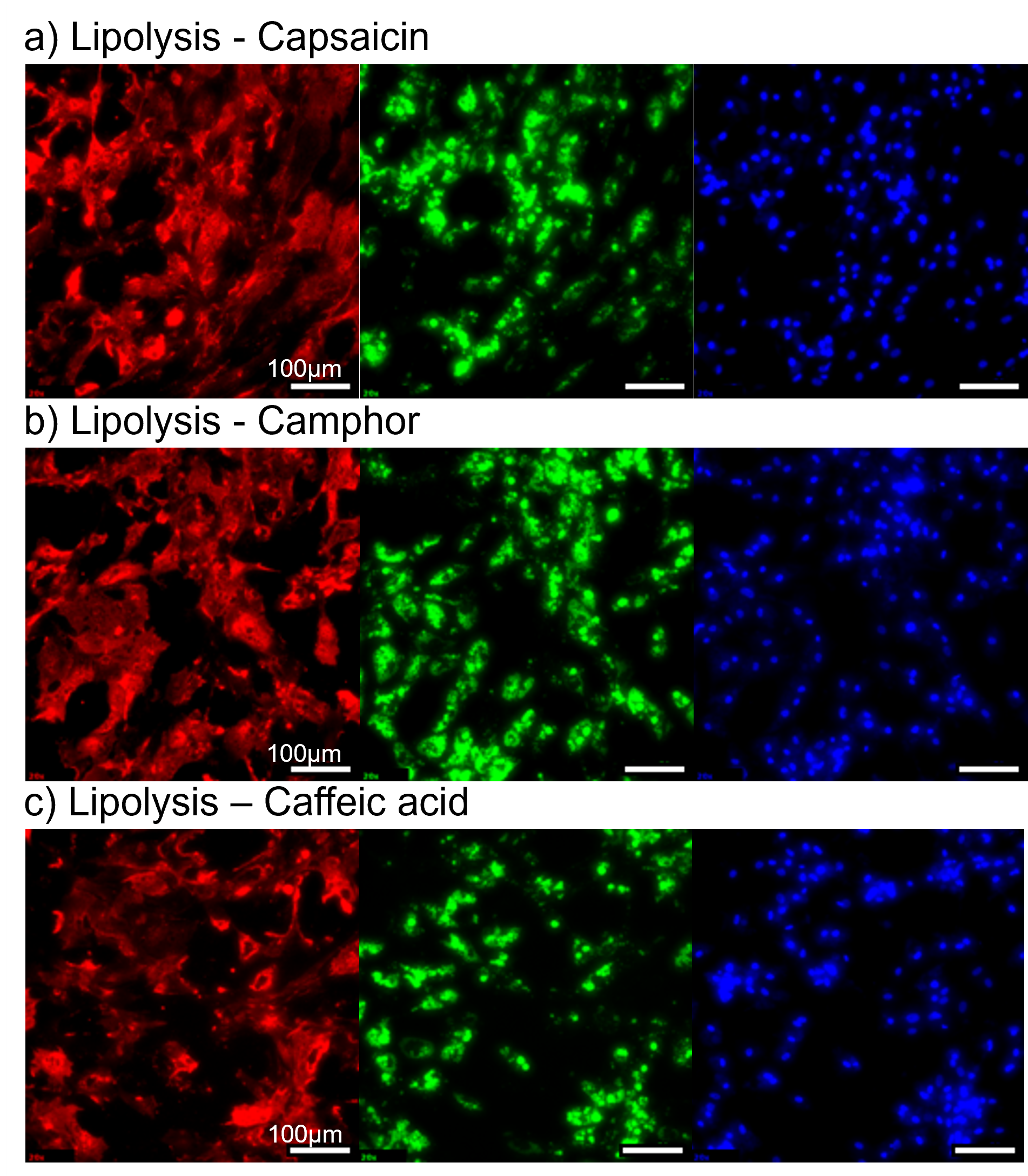


**Figure S2.** Immunofluorescence staining of SGBS adipocytes treated with capsaicin, camphor and caffeic acid and their combinations from day 12 to day 14 of differentiation (lipolysis phase). Green, BODIPY 493,503 for neutral lipids. Red, TRPV1 immunofluorescence with Alexa Fluor 647 secondary. Blue, DAPI nuclei. Figure represents separated fluorescence channels that correspond to Figure 4 a in main manuscript.


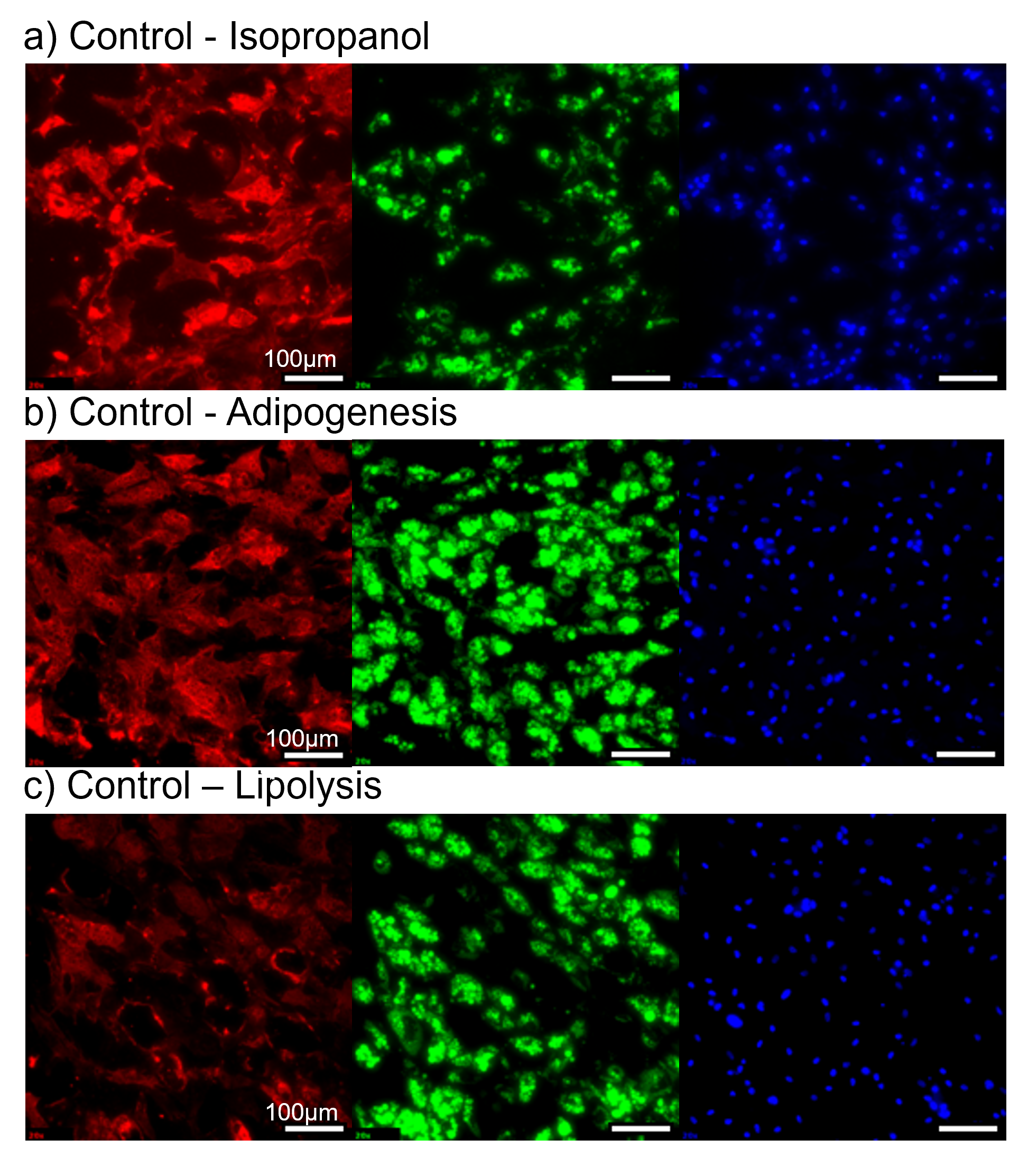


**Figure S3.** Immunofluorescence staining of SGBS adipocytes controls including isoproterenol treatment as a positive control for lipolysis), and untreated cells for adipogenesis and lipolysis. Green, BODIPY 493,503 for neutral lipids. Red, TRPV1 immunofluorescence with Alexa Fluor 647 secondary. Blue, DAPI nuclei. Figure represents separated fluorescence channels that correspond to Figure 4 a in main manuscript.

**Table S1**. Primer sequences used for gene expression analysis in SGBS cells

| Gene | Forward | Reverse |
| --- | --- | --- |
| B-actin | 5'-GACCCAGATCATGTTTGAGACC-3' | 5'-CATCACGATGCCAGTGGTAC-3' |
| RPLP0 | 5-GCAATGTTGCCAGTGTCTG-3′ | 5′-GCCTTGACCTTTTCAGCAA-3′ |
| ATGL | 5′-CCCACTTCAACTCCAAGGACGA-3′ | 5′-GCAGGTTGTCTGAAATGCCACC-3′ |
| PGC lα | 5'-TTCCTCTGACCCCAGAGTCACC-3' | 5'-TTGCAAGAGGACTTCAGCTTTGG-3' |
| ADIPOQ | 5'-TTAAAACCTCCCCCAAGCAGA-3' | 5'-GCCTTGAGGAACAGGGATGAG-3' |
| FABP4 | 5'-GGAAAGTCAAGAGCACCATAACC-3' | 5'-GCTCTCTCATAAACTCTCGTGGAAG-3' |
| PLIN1 | 5'-GAACAAGTTCAGTGAGGTAGCAGC-3' | 5'-CTTGGTTGAGGAGACAGCAGG-3' |
| HSL | 5'-GAAGCCTTTGAGATGCCACTG-3' | 5'-CTCACTGTCCTGTCCTTCACG-3' |
| PPARγ | 5'-GTGGCCGCAGATTTGAAAGAAG-3' | 5'-CCATGGTCATTTCGTTAAAGGCIG-3' |
| CEBPα | 5′-AGCCTTGTTTGTACTGTATG-3′ | 5-AAAATGGTGGTTTAGCAGAG-3′ |

**Table S2.** Network metrics and gene identifiers for camphor targets

| Gene name | Betweenness | Closeness | Degree | log score | Ensembl Gene ID | Uniprot ID |
| --- | --- | --- | --- | --- | --- | --- |
| FABP4 | 125.81 | 0.51 | 8 | -0.58 | ENSG00000170323 | P15090 |
| PPARA | 258.32 | 0.6 | 13 | -0.72 | ENSG00000186951 | Q07869 |
| P2RX4 | 72.8 | 0.45 | 7 | -4.18 | ENSG00000110955 | Q99571 |
| PPARG | 109.05 | 0.57 | 10 | -3.86 | ENSG00000132170 | P37231 |
| POR | 32.83 | 0.41 | 6 | -4.17 | ENSG00000186088 | P16435 |
| NOS2 | 112.01 | 0.5 | 8 | -0.53 | ENSG00000105379 | P35228 |
| NOS1 | 45.54 | 0.45 | 6 | -4.21 | ENSG00000120329 | P29475 |
| P2RX6 | 57.93 | 0.45 | 6 | -4.15 | ENSG00000155849 | O15547 |
| NR1H4 | 64.67 | 0.47 | 7 | -0.44 | ENSG00000118017 | Q96RI1 |
| VDR | 79.91 | 0.48 | 7 | -0.54 | ENSG00000111424 | P11473 |

**Table S3.** Network metrics and gene identifiers for capsaicin targets

| Gene name | Betweenness | Closeness | Degree | log score | Ensembl Gene ID | Uniprot ID |
| --- | --- | --- | --- | --- | --- | --- |
| HIF1A | 138.24 | 0.58 | 11 | -1.25 | ENSG00000100644 | Q16665 |
| STAT3 | 51.07 | 0.55 | 11 | -0.44 | ENSG00000168610 | P40763 |
| MTOR | 41.8 | 0.49 | 11 | -0.39 | ENSG00000198793 | P42345 |
| FASN | 232.36 | 0.54 | 10 | -0.47 | ENSG00000169710 | P49327 |
| PIK3CD | 11.48 | 0.49 | 10 | -4.65 | ENSG00000171608 | O00329 |
| ATM | 45.01 | 0.51 | 10 | -4.86 | ENSG00000149311 | Q13315 |
| ESR1 | 120.49 | 0.53 | 12 | -1.04 | ENSG00000091831 | P03372 |
| PRKDC | 67.24 | 0.56 | 10 | -4.48 | ENSG00000149483 | P78527 |
| ESR2 | 86.31 | 0.53 | 10 | -1.7 | ENSG00000140009 | Q92731 |

**Table S4.** Network metrics and gene identifiers for caffeic acid targets

| Gene name | Betweenness | Closeness | Degree | log score | Ensembl Gene ID | Uniprot ID |
| --- | --- | --- | --- | --- | --- | --- |
| STAT3 | 216.44 | 0.63 | 14 | -0.71 | ENSG00000168610 | P40763 |
| NOS2 | 124.05 | 0.57 | 10 | -0.38 | ENSG00000105379 | P35228 |
| JAK2 | 9.11 | 0.47 | 5 | -3.37 | ENSG00000096968 | O60674 |
| NFE2L2 | 134.05 | 0.51 | 8 | -0.66 | ENSG00000116044 | Q16236 |
| ESR2 | 73.28 | 0.57 | 10 | -1.54 | ENSG00000140009 | Q92731 |
| ESR1 | 149.42 | 0.57 | 11 | -0.76 | ENSG00000091831 | P03372 |
| PTPN1 | 45.48 | 0.55 | 8 | -0.43 | ENSG00000196363 | P18031 |
| PTGS2 | 56.92 | 0.5 | 6 | -0.52 | ENSG00000073756 | P35354 |
| RBM39 | 17.12 | 0.48 | 6 | -3.99 | ENSG00000136518 | Q14498 |
| NOS3 | 102.74 | 0.48 | 6 | -3.1 | ENSG00000164867 | P29474 |
